# Structural Changes Observed in the Piriform Cortex in a Rat Model of Pre-motor Parkinson’s Disease

**DOI:** 10.3389/fncel.2018.00479

**Published:** 2018-12-10

**Authors:** Marco Sancandi, Emma Victoria Schul, Georgia Economides, Andrew Constanti, Audrey Mercer

**Affiliations:** UCL School of Pharmacy, London, United Kingdom

**Keywords:** olfaction, interneurons, extracellular matrix, perineuronal nets, early stage Parkinson’s disease, GLP-1 agonist, exendin-4

## Abstract

Early diagnosis of Parkinson’s disease (PD) offers perhaps, the most promising route to a successful clinical intervention, and the use of an animal model exhibiting symptoms comparable to those observed in PD patients in the early stage of the disease, may facilitate screening of novel therapies for delaying the onset of more debilitating motor and behavioral abnormalities. In this study, a rat model of pre-motor PD was used to study the etiology of hyposmia, a non-motor symptom linked to the early stage of the disease when the motor symptoms have yet to be experienced. The study focussed on determining the effect of a partial reduction of both dopamine and noradrenaline levels on the olfactory cortex. Neuroinflammation and striking structural changes were observed in the model. These changes were prevented by treatment with a neuroprotective drug, a glucagon-like peptide-1 (GLP1) receptor agonist, exendin-4 (EX-4).

## Introduction

Parkinson’s disease (PD) is a highly debilitating neurodegenerative disorder associated with degeneration of the nigrostriatal dopaminergic pathway, with no present cure. PD is now thought to be, in the vast majority of cases, the result of an interplay between a genetic pre-disposition, neuroinflammation, and very probably, some form of neurotoxicity (Athauda and Foltynie, 2015; Maiti et al., 2017; Shihabuddin et al., 2018 for review). However, the cause and mechanisms behind the progression of PD remain unclear. The staging procedure described by Braak et al. (2003, 2004) and Braak and Del Tredici (2017) reported a neurone-to-neurone spread of α-synuclein-containing bodies (Lewy pathology – LP) from the enteric nervous system and olfactory system to the central nervous system (CNS) that was correlated with the appearance and severity of both motor and non-motor symptoms of PD. The reliability of this staging system, however, has been challenged over the years with evidence suggesting that the staging of LP may be governed by a combination of selective vulnerability and a possible lack of compensatory mechanisms in place in regions with low synaptic connectivity (Halliday and McCann, 2009; Hunn et al., 2015; Surmeier et al., 2017; proposed by “threshold” theory: Engelender and Isacson, 2017; Rietdijk et al., 2017; Jellinger, 2018) rather than the entry of pathogens *via* the olfactory bulb (OB) (Rey et al., 2018 for review). Nevertheless, there is an agreement that olfactory structures, including the OB, anterior olfactory nucleus (AON) and piriform cortex (PC), are affected in a prodromal phase in which the appearance of hyposmia, a non-motor symptom, precedes the first signs of motor dysfunctions by several years (Fullard et al., 2017; Schapira et al., 2017). As motor function is the consequence of neuronal loss of dopaminergic neurones in the substantia nigra pars compacta (SNc), the most common treatment for PD is temporary replacement of brain dopamine (DA), transient reactivation of DA function, providing a temporary, though imperfect restoration of movement (Stocchi et al., 2008; Pahwa and Lyons, 2009; Maiti et al., 2017 for review). However, the pathology of PD encompasses a much wider neurological basis and range of non-motor symptoms (NMS), including hyposmia, that cannot be accounted for by DA loss alone and are linked to depletion of other key brain neurotransmitters such as noradrenaline (NA), 5-hydroxytryptamine (5-HT), acetylcholine (ACh) and γ-aminobutyric acid (GABA) (Hou and Lai, 2007; Delaville et al., 2011; Doty, 2012, 2017; Jellinger, 2015; Błaszczyk, 2016; Schapira et al., 2017). The degree of involvement of NMS in the progression of the disease is still poorly understood and clear insights on their neuropathobiology (focussing on NMS), that are features of prodromal PD, like the loss of smell, are needed. Early involvement of olfactory structures in early PD, mainly OB, and AON, has been studied extensively (Ross et al., 2008; Doty, 2012; Schapira et al., 2017; Rey et al., 2018), however, little is known about the pathology in the olfactory cortex despite the known presence of dopaminergic and noradrenergic inputs from the SNc and locus coeruleus (LC) into this region (Fallon and Moore, 1978). In view of these considerations, a rat neurotoxin-based model of pre-motor PD presenting a partial reduction of both DA and NA levels and displaying hyposmia and cognitive deficits with absence of motor symptoms was used to determine potential structural and cellular changes in the PC in the early stage of the disease. Of particular interest was the study of the effect of partial DA and NA denervation on this region, the presence of neuroinflammation following neuronal degeneration, the distributions of GABAergic interneurons, that have been shown to be affected in olfactory pathways of both genetic PD models and PD patients (Ubeda-Banon et al., 2010; Doty, 2012), and the integrity of perineuronal nets (PNNs), a component of the extracellular matrix (ECM) enveloping these neurones (Alpár et al., 2006; Ajmo et al., 2008). Although a disruption of the PNNs appears to be at the root of many neurological disorders (Baig et al., 2005; Hobohm et al., 2005; Berretta et al., 2015), their involvement in the early stages of PD remains to be determined.

As non-motor symptoms, particularly hyposmia, emerge many years before diagnosis, the potential window of opportunities to prevent or slow the progression of the disease has led to an increased research activity to explore novel targets and find new therapeutic drugs. One such drug, exendin-4 (EX-4), a glucagon-like peptide-1 (GLP-1) receptor agonist, currently in clinical use for type II diabetes, has demonstrated neuroprotective effects in several animal models of PD (Bertilsson et al., 2008; Harkavyi et al., 2008; Kim et al., 2009, 2017; Li et al., 2009; Cao et al., 2016; Chen et al., 2018; Yun et al., 2018). These data, together with its excellent safety profile (Bertilsson et al., 2008; Harkavyi et al., 2008; Kim et al., 2009; Li et al., 2009; MacConell et al., 2015; Cao et al., 2016; Yun et al., 2018), even at high doses (Rocha-Ferreira et al., 2018) and its ability to pass the blood brain barrier (Kastin and Akerstrom, 2003; Athauda et al., 2017a; Zanotto et al., 2017), supported its use in clinical trials in patients with PD (Aviles-Olmos et al., 2013, 2014; Athauda et al., 2017a). Patients that received EX-4 showed improvements in motor symptoms, cognition, depression and sleep quality compared with patients treated with conventional PD medication (Aviles-Olmos et al., 2013, 2014). EX-4 also improved off-medication motor scores after 48 weeks’ exposure and 12 weeks wash-out in PD patients compared with placebo (Athauda et al., 2017a,b). However, the effect of EX-4 on the underlying pathophysiology in PD patients remains unclear. The protective effects of EX-4 (Athauda and Foltynie, 2016a,b), the known presence of GLP-1 receptors (GLP-1Rs) in the PC (Cork et al., 2015) and the potential role of these receptors in olfaction (Llewellyn-Smith et al., 2011; Cork et al., 2015; Trapp and Cork, 2015) led us to study the effect of EX-4 on the structural changes observed in the olfactory cortex of the pre-motor model and the associated olfactory and behavioral consequences of the dual toxin treatment.

## Materials and Methods

### Animals

All procedures used throughout this study were carried out according to the British Home Office regulations with regard to the Animal Scientific Procedures Act 1986. Male Wistar rats weighing 200–250 g (Harlan Laboratories, Inc., United Kingdom) were kept under constant conditions of humidity (40–60%), temperature (18–22°C) and a 12 h light-dark cycle.

### Stereotaxic Surgery and Drug Administration

**FIGURE 1:**
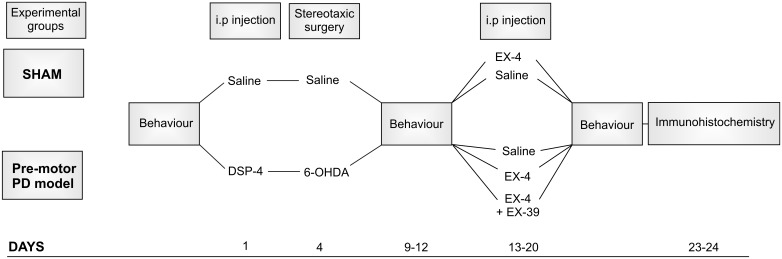
Protocol for dual neurotoxin administration in rats to generate a pre-motor PD model. Behavioral testing (sucrose preference test, rotarod, novel object recognition, hidden food test, habituation/dishabituation test) was carried out before, after toxins and after the 7-day treatment with exendin-4 (EX-4). The study included five experimental groups: sham, sham + EX-4, 6-OHDA + DSP-4 (model), 6-OHDA + DSP-4 + EX-4 (model + EX-4) and 6-OHDA + DSP-4 + EX-4 + EX9-39 (model + EX-4 + EX9-39). Histological procedures occurred after animals were culled at days 23–24.

The protocol used for toxin administration is presented in Figure 1. Intraperitoneal administration of the noradrenergic neurotoxin N-(2-chloroethyl)-N-ethyl-2-bromobenzylamine (DSP-4, Sigma-Aldrich) at a dose of 25 mg/kg, was performed 4 days prior to the dopaminergic neurotoxin insult with 6-hydroxydopamine (6-OHDA). This injection was carried out to induce partial degeneration of noradrenergic cells in the CNS, mainly those in the LC (Supplementary Figure S1; Ross and Stenfors, 2015). Previous studies showed that high doses of DSP-4 (50 mg/kg) may result in an increase in neophobia, defensive behavior, an increased aggressiveness and an alteration of danger perception (Delini-Stula et al., 1984; Harro et al., 1995). However, the low dose of DSP-4 (25 mg/kg) used in this study did not result in any adverse effects or change in behavior patterns (no increased grooming activities, number of stools, no difference in behavioral pattern following open field or sucrose preference tests). Stereotaxic surgery was then performed to administer bilateral injections of either 6-OHDA (Sigma-Aldrich – dissolved in saline solution containing 0.9% ascorbic acid) or saline containing 0.9% ascorbic acid for controls. The optimum doses for DSP-4 and 6-OHDA were chosen to induce partial reduction of NA and DA levels, respectively, based on previous studies (Jonsson et al., 1981; Przedborski et al., 1995), mimicking the early stage of the disease. Animals were anaesthetized using isoflurane (5% v/v in O_2_ for induction and 2% v/v in O_2_ for maintenance) delivered through a fitted nose mask and rats were secured to a stereotaxic frame using blunt ear bars (David Kopf, United States). Each animal received 15 μg of 6-OHDA per striatum (or vehicle) at a flow rate of 1 μL/min^-1^ to induce partial destruction of the nigro-striatal dopaminergic system. The following coordinates from the atlas of Paxinos and Watson (1982) were used to locate the ventrolateral area of the dorsal striatum, from Bregma: AP +1.0 mm, ML +3.0 mm, DV -6.5 mm. Animals were closely monitored for a week following the surgical procedures. EX-4 (Sigma-Aldrich) was administered twice daily via intraperitoneal injection (with saline as a vehicle) at a dose of 0.5 μg/kg for a period of 7 days, one week after stereotaxic surgeries. The GLP-1R antagonist EX9-39 (Sigma-Aldrich – 2 μg/kg with saline as a vehicle) was injected prior to EX-4 injection twice daily for 7 days.

In this study, rats were divided into five experimental groups (Figure 1): sham (*n* = 10), sham + EX-4 (*n* = 10), model (*n* = 10), model + EX-4 (*n* = 10) and model + EX-4 + EX9-39 2 μg/kg (*n* = 5).

### Behavioral Experiments

All experimental groups were subjected to behavioral tests prior to the start of the procedures, 5–7 days after stereotaxic injections of 6-OHDA and after the 7-day treatment with either saline, EX-4 or EX-4 + EX9-39 (see Figure 1). Rats were subdivided into groups that underwent only one behavioral test [sucrose preference test, Novel Object Recognition (NOR) test, or rotarod] to prevent confounding results. However, all animals were assessed for hyposmia with two tests: the buried food test and habituation/dishabituation test. All data are represented as mean ± standard error of the mean (SEM).

Rats were habituated in the testing arena twice for 5 min the day before the buried food test and fasted overnight. Animals were then placed in the same environment containing a “treat” (banana chips) hidden under the sawdust and the time taken to locate the treat was assessed. Animals were given 5 min to locate the treat and those unable to find it during this period were given a score of 300 s. Five animals per experimental group were also tested with the treat in view. The habituation/dishabituation test was used to determine whether the animals were able to detect and differentiate different odors as previously described (Arbuckle et al., 2015). Rats were habituated in the testing arena for 30 min. Each animal was then exposed to an odor or water (for control) on a cotton swab for 2 min in three consecutive trials with a minute between trials and the total time spent exploring the cotton swab was measured. Both non-social (paprika and vanilla) and social odors were used in this test. Two social odors (social 1 and social 2) were obtained by wiping a cotton swab across the bottom of two dirty cages that contained an equal number of male rats.

Level of anxiety was tested using a sucrose preference test. Rats were first exposed to a 4-day habituation phase in which they were provided with two bottles, one containing water and the other containing a 1% sucrose solution. After the habituation phase, the animals were individually housed and sucrose consumption was measured over a two-day period by weighing the bottles daily. Sucrose preference was calculated as the percentage of total sucrose solution consumed compared with total fluid intake.

Cognition was measured using a Novel Objection Recognition (NOR) test. Rats were placed in the testing arena for a period of 5 min for 2 days prior to testing. The task consisted of three phases: habituation, familiarization and test phase. Animals were first allowed to explore the testing arena freely, in the absence of objects, for 10 min. Animals were then removed from the arena and placed in their holding cages. Rats were then reintroduced into the arena containing two identical objects for a period of 5 min. One hour later, rats were returned to the arena that contained one familiar and one novel object for 5 min. A discrimination ratio (DR) was calculated by dividing the time spent exploring the novel object by the total exploration time.

Motor coordination was assessed using a Rotarod Test. Animals were placed on an accelerating rotating rod (drop height 30 cm) and the speed at which the animals fell off was noted. Rats were subjected to a training period of three consecutive days prior to the testing day. On the first day of training, animals were placed on a rod rotating at a constant slow speed in three consecutive trials. On the second and third training day, animals were placed on the rod before starting acceleration (start speed of 4 rpm, acceleration rate 9 rpm/min) until they fell off in three consecutive trials. On the day of testing, animals were placed on the accelerating rod (start speed of 4 rpm, acceleration rate 9 rpm/min) and the time of fall was recorded.

### High Performance Liquid Chromatography (HPLC)

Dissected rat brain tissues (SNc, striatum and PC) were weighed and homogenized in appropriate volumes of homogenizing solution (0.1 M perchloric acid containing 400 mM sodium metabisulphite) and then microcentrifuged. Determination of DA and NA levels was performed with a Jasco PU-980 pump HPLC coupled to electrochemical detection (Coulochem II-ESA model 5011 analytical cell), equipped with a Capital Hypersil column (250 mm x 4.6 mm id 5 μm) using a mobile phase (0.01 M sodium dihydrogen orthophosphate dehydrate, 0.9 mM 1-octanesulfonic acid sodium salt, 0.1% dibutylamine, 12.5% methanol, pH 3.2) at a flow rate of 1 ml min^-1^. Concentrations of the monoamines were expressed as ng/g ± SEM and were calculated by reference to an internal standard, dihydroxybenzylamine (DHBA – 0.5 ng). Data were captured using Antec’s Scientific Clarity software.

### Immunohistochemistry

#### Neuronal Distributions

All histological procedures used in this study have been described previously (Hughes et al., 2000). Brain slices were obtained from rats that were anaesthetized by inhalation of isoflurane and intraperitoneal injection of Euthatal (Merial, Harlow, United Kingdom) (60 mg/kg) and then perfused transcardially with ice-cold oxygenated artificial cerebrospinal fluid (ACSF) containing in mM: 124 NaCl, 25.5 NaHCO_3_, 3.3 KCl, 1.2 KH_2_PO_4_, 1 MgSO_4_, 2.5 CaCl_2_, 15 mM D-Glucose equilibrated with 95% O_2_/5% CO_2_. Brains were then removed and fixed overnight (4% paraformaldehyde, 0.2% saturated picric acid solution, 0.025% glutaraldehyde solution in 0.1 M Phosphate buffer). Fifty micrometers parasagittal sections containing the piriform cortex were cut with a vibratome (Agar Scientific). One in 12 sections were collected and 3–4 slices per animal were used per staining. Sections were incubated first in 1% H_2_O_2_ for 30 min and then in 1% sodium borohydride (NaBH_4_) for 30 min to decrease background staining and then in either 1% bovine serum albumin for the Biotinylated Wisteria floribunda lectin (WFA) staining used to label PNNs or in 10% normal goat serum (NGS) for all other antibodies for another 30 min to block non-specific antibody binding. Sections were incubated overnight at 4^o^C in a mixture of primary antibodies and triton X-100 (Sigma-Aldrich) (1% Triton for GAD-67 – 0.1% for all other antibodies) made up in phosphate buffer solution. Primary antibodies used in this study and antibody specificity are listed in Table 1. Sections for fluorescence microscopy were then incubated for 2 h in a mixture of fluorescently labeled secondary antibodies, anti-mouse fluorescein isothiocyanate (FITC) and goat anti-rabbit Texas Red (TR) made up in PBS. All fluorescently labeled sections were then mounted on slides in Vectashield (Vector Laboratories) and studied using a confocal microscope (Zeiss LSM 710). Six to seven Z-stacks from the piriform cortex of each slice in each animal (1 in 15 PC sections – 3–4 slices per animal) were obtained using a 10× objective and immunopositive cell bodies were counted using a Cell Counting plug in ImageJ software. Cellular densities were expressed as the number of cells per mm^3^± SEM.

**Table 1:** List of primary antibodies used in this study.

Antibody	Immunogen	Manufacturer/Investigator	Species	Catalog/lot number	Dilutions
Parvalbumin	Purified frog muscle parvalbumin	Sigma	Rabbit	P3088 Clone PARV-9 Lot #048K4752	1:1000
CCK	Synthetic human gastrin/CCK 2-17 conjugated with carbodiimide to keyhole limpet hemocyanin	Cure Digestive Diseases Research Center, UCLA	Mouse	#9303	1:1000
Calretinin	Recombinant rat calretinin	Millipore	Rabbit	AB5054/LV1532272 Lot #2049207	1:1000
Calbindin	Recombinant chick CaBP	Gift from Dr. K. Baimbridge (2000)	Rabbit	R9501	1:1000
Somatostatin	Somatostatin-14 conjugated to keyhole limpet hemocyanin	Gift from Dr. A. Duchan (2000)	Mouse		1:3000
NPY	Neuropeptide Y coupled with bovine thyroglobulin with glutaraldehyde	Immunostar	Rabbit	22940 Lot #1112001	1:1000
VIP	Synthetic porcine VIP conjugated to bovine thyroglobulin with carbodiimide linker	Immunostar	Rabbit	20077 Lot#1129001	1:1000
GAD67	Synthetic peptide from mouse GAD67 (amino acids (87-106)	Sigma	Mouse	MAB 5406 Lot #2549419	1:1500
TH	SDS-denatured rat tyrosine hydroxylase purified from pheochromocytoma	Sigma	Rabbit	T8700-1VL Lot #SLBL8773V	1:7500
GFAP	Purified glial filament	Millipore	Mouse	MAB 3402 #2549419	1:7500
Iba1	C-terminus of Iba1	Wako	Rabbit	019-19741	1:1000


#### Neuroinflammation Staining

Following incubation in primary antibodies, sections for immunoperoxidase staining (glial fibrillary acidic protein (GFAP) and ionized calcium-binding adapter molecule 1 (Iba1) staining) were incubated overnight in secondary antibodies, biotinylated goat anti-mouse or anti-rabbit antibody (1:500, Vector Laboratories) made up in PBS. To visualize the stained neurones, sections were incubated first in ABC (Vector Laboratories) for 2 h and then in 3,3′-diaminobenzidine (DAB – Sigma-Aldrich). H_2_O_2_ was added to the DAB solution to allow the reaction until the filled cells were sufficiently labeled. Sections from the different experimental groups (1 in 12 PC sections – 4 slices per animal) were processed together using the same immunoreagents and the DAB reaction was stopped at the same time to allow comparison between groups. Sections were then placed onto Superfrost slides, dehydrated, cleared with Histoclear and mounted using DPX (Sigma-Aldrich). Levels of GFAP and Iba1 immunohistochemical staining were measured by quantitative thresholding image analysis as previously described (Rahim et al., 2012). Four non-overlapping images of the piriform cortex in each section were captured using a DMR microscope and Leica Application Suite V4 (Leica Microsystems) at 10× magnification with constant light intensity, microscope calibration and video camera settings. Image-Pro Premier (Media Cybernetics, Cambridge, United Kingdom) was used to analyze the images and measure immunoreactivity using a constant threshold that was applied to all images for each respective antigen. Data are presented as the mean percentage area of immunoreactivity ± SEM. 3D reconstructions of Iba1-immunopositive cells (*n* = 8 per experimental group) were obtained with a Neurolucida software (MBF Bioscience) and morphological characteristics of these cells were analyzed using Sholl and branched structure analyses. Data are represented as mean ± standard deviation (SD).

##### Tyrosine Hydroxylase (TH) staining

TH immunostaining was carried out on coronal sections containing SNc and ventral tegmental area (VTA) according to the immunoperoxidase protocol described above. One in four slices per animal were collected and processed. Sections were dehydrated and mounted using DPX. The optical fractionator probe was used to determine the number of TH-immunopositive neurons in the SNc and VTA (Stereo Investigator, MicroBrightField) using a Nikon microscope coupled to a computer-controlled x-y-z motorized stage and an MBF video camera system. Unbiased stereology was carried out on five slices per animal (*n* = 6 per experimental group) with the following parameters: counting frame 60 μm × 60 μm; grid size 200 μm × 200 μm; section thickness 50 μm, dissector height 12 μm. Only neurones with visible nuclei and dendrites were counted. Data are displayed as cell density per mm^3^ ± SEM.

### Statistics

All data in this study were analyzed using IBM SPSS Statistics Version 22.0. The Shapiro–Wilk test and the Kolmogorov–Smirnov test were carried out prior to statistical analysis to determine whether the data followed a normal distribution. The parametric one-way ANOVA, repeated measured ANOVA or unpaired *t*-test were then used when the data followed a normal distribution and the non-parametric Kruskal–Wallis test were use in case of a non-normal distribution.

## Results

### Effect of DSP-4 and Bilateral 6-OHDA Injections on Brain Dopaminergic and Noradrenergic Levels in the Pre-motor PD Model

**FIGURE 2:**
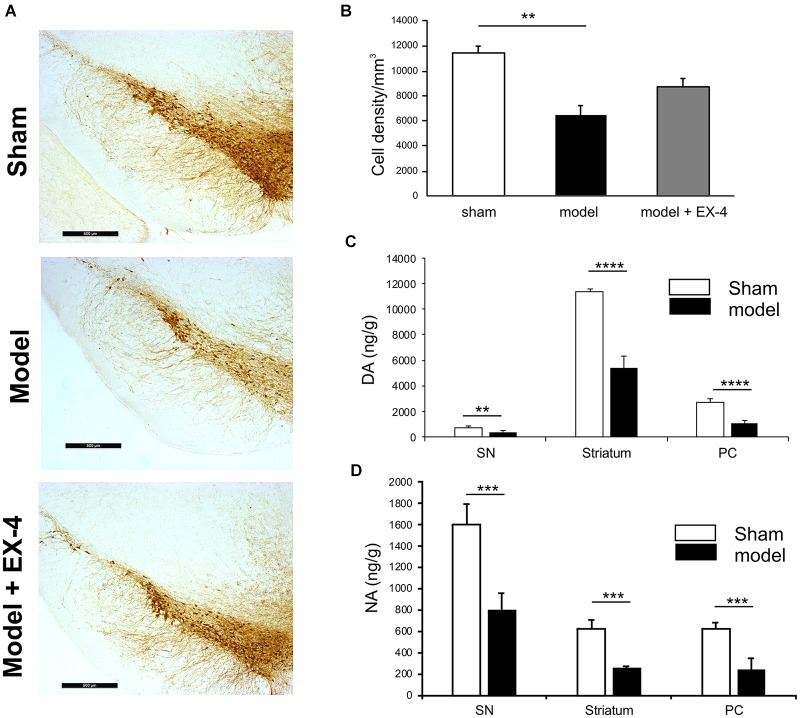
Reduction of dopamine and noradrenergic levels in a model of pre-motor PD. **(A)** Representative images of TH-staining in the substantia nigra pars compacta (SNc) in the sham-operated animals (top panel), pre-motor model (middle panel), and model treated with EX-4 (bottom panel). Scale bars represent 500 μm. **(B)** Number of TH-positive cells in the SN expressed as cell density per mm^3^ (*n* = 6 animals per experimental group). A decrease in the number of TH-positive cells was observed in the pre-motor model (unpaired *t*-test ^∗∗^*P* < 0.01). This decrease was partially prevented by treatment with EX-4 (unpaired *t*-test P_sham_ _vs._ _model__+__EX-4_ >0.05; P_model_ _vs.model__+__EX-4_ >0.05). **(C)** HPLC measurements of tissue levels of dopamine (DA) in SN, striatum and piriform cortex (PC) expressed as ng/g wet weight. Levels of DA were reduced in the three regions (unpaired *t*-test *n* = 4–5 animals per group ^∗∗^*P* < 0.01, ^∗∗∗∗^*P* < 0.0001). **(D)** HPLC measurements of tissue levels of noradrenaline (NA) in SN, striatum and piriform cortex (PC) expressed as ng/g wet weight. Levels of NA were reduced in the three regions (unpaired *t*-test *n* = 4–5 animals per group ^∗∗∗^*P* < 0.001).

Immunohistochemical staining of TH cells in the SNc (Figure 2A) and unbiased stereology (Figure 2B) revealed that dopaminergic lesions in the pre-motor model significantly decreased TH immunoreactivity by 43.5 ± 5.3% in the SNc compared with that observed in sham animals (*P* < 0.01). The toxin injections had no effect on the number of TH-positive cells in the VTA (Supplementary Figure S2). Injections of EX-4 in the pre-motor model partially prevented the neuronal loss in the SNc (Figures 2A,B). Levels of DA and NA, measured by HPLC in the SNc were significantly reduced by 47.6 ± 2.4 and 49.9 ± 1.6%, respectively, in the pre-motor model compared with those measured in the sham animals (unpaired *t*-test, *P* < 0.01) (Figures 2C,D). HPLC measurements of DA and NA in the piriform cortex revealed a reduction of 39.8 ± 1.1 and 40 ± 1.4%, respectively, in the models compared with those in the shams (unpaired *t*-test, *P*_DA_ < 0.0001, *P*_NA_ < 0.001) (Figures 2C,D).

### The Pre-motor PD Model Did Not Display Anhedonia or Motor Symptoms

**FIGURE 3:**
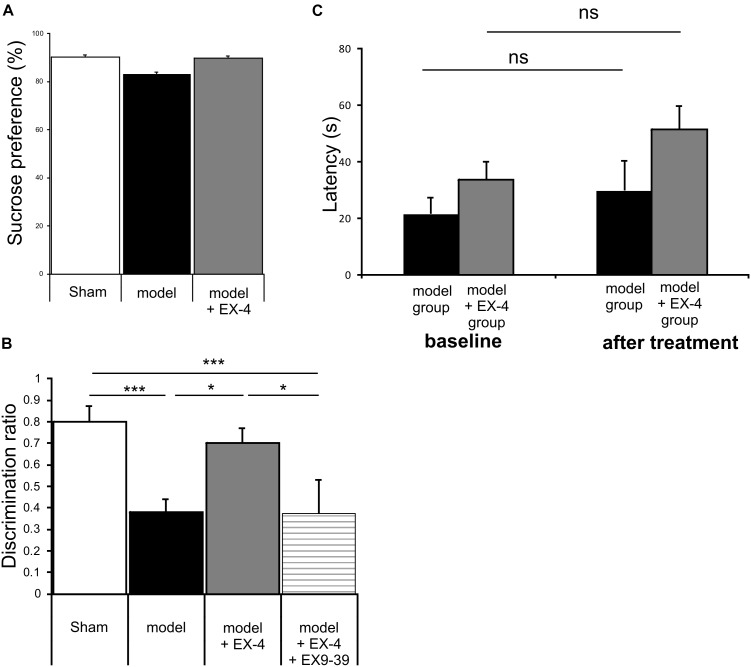
The pre-motor PD model displayed cognitive deficits that were prevented by treatment with EX-4, in the absence of anhedonia or motor dysfunction. **(A)** At 18 days after surgery, the pre-motor model did not display anhedonia, as the percentage of sucrose consumed was similar in all experimental groups (one-way ANOVA test *n* = 4–5 animals per experimental group – *P* > 0.05). Data are presented as mean % sucrose preference ± SEM. **(B)** Locomotion in the pre-motor model receiving either saline (*n* = 5) or EX-4 (*n* = 4) treatments was tested at baseline and after treatment with either saline (model) or EX-4 (model + EX-4). Data are presented as the mean latency (s) at which the animals fell from the rotating rod ± SEM. The latency after treatment in both groups (model and model + EX-4 – Day 18) was similar to that observed at baseline (prior to DSP-4 injection) (one-way ANOVA with repeated measures; ns *P* > 0.05). **(C)** Cognitive deficits, measured as a decrease in the discrimination ratio (DR) using a novel object recognition (NOR) test, were observed in the pre-motor model. EX-4 prevented this deficit and the addition of the GLP-1R antagonist EX9-39 inhibited this effect (one-way ANOVA with Gabriel’s method *post hoc* analysis *n* = 4–5 animals per experimental groups ^∗^*P* < 0.05, ^∗∗∗^*P* < 0.001).

Animals were subjected to behavioral tests to validate the presence of non-motor symptoms and absence of motor symptoms in the pre-motor PD model. These tests were carried out at baseline, 7 days after surgery and 18 days after surgery (See Figure 1 for protocol). The level of anhedonia in all experimental groups was investigated using the sucrose preference test. One-way ANOVA revealed that groups did not show any statistically significant differences at 7 days (data not shown) or at 18 days after surgery [*F*_(7,24)_ = 0.479, *P* > 0.05] (Figure 3A). Motor function was also not affected in all experimental groups at 18 days after surgery (one-way ANOVA with repeated measures, *P* > 0.05) (Figure 3B).

### The Cognitive Deficit Displayed by the Pre-motor PD Model Was Prevented by Treatment With EX-4

Cognitive deficits were studied by comparing the discrimination ratio (DR) score of all experimental groups over time (*n* = 4–5 Figure 3C). The DR score decreased over time across groups [two-way ANOVA with repeated measures – *F*_(2,39)_ = 23.784, *P* < 0.05] and a statistically significant interaction effect between the DR over time and experimental group was observed [*F*_(14,39)_ = 3.257, *P* < 0.001]. Cognitive deficits (indicated by a decreased DR) were observed in the pre-motor model at 18 days after surgery (Figure 3C) and these deficits were already present one week after surgery (data not shown). Treatment with EX-4 prevented the cognitive deficit in the model and this effect was mediated by GLP-1R activation as it was blocked by co-administration of the competitive GLP-1R antagonist, EX9-39 (Figure 3C).

### Hyposmia Displayed by the Pre-motor PD Model Was Prevented by EX-4 Treatment

**FIGURE 4:**
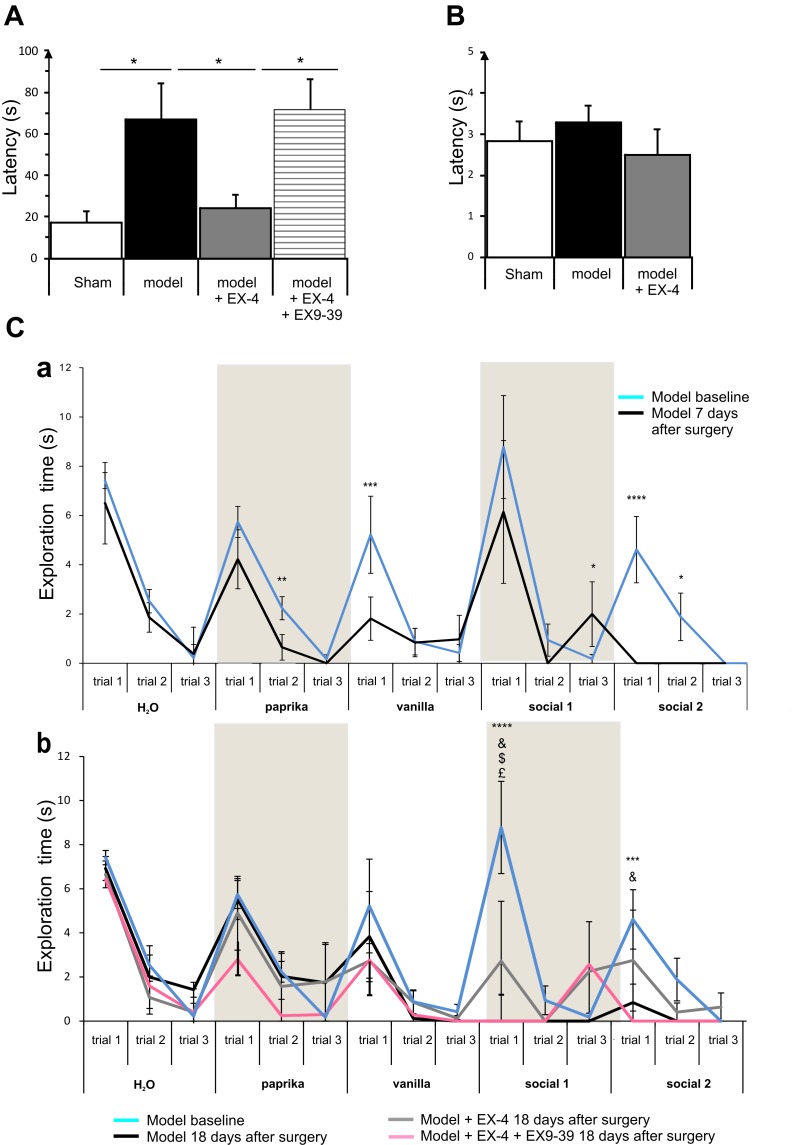
The pre-motor PD model displayed hyposmia that was prevented by treatment with EX-4. **(A)** Hyposmia was tested using the hidden food test (sham *n* = 10; model *n* = 10; model + EX-4 *n* = 10; model + EX-4 + EX9-39 *n* = 5). Data are represented as mean ± SEM. The latency time (s) time taken by the pre-motor model animals to find the hidden treat was increased compared with that by the sham animals. Hyposmia was prevented by treatment with EX-4 and this effect was inhibited when the animals received the GLP-1R antagonist EX9-39 (one-way ANOVA with Gabriel’s method *post hoc* analysis ^∗^*P* < 0.05). **(B)** Open food test. Animals were tested with the treat in view and the latency time (s) in finding the treat was similar in all experimental groups (unpaired *t*-test *n* = 5 animals per experimental group *P* > 0.05). **(C)** Olfactory deficits were also tested using the habituation/dishabituation test at baseline, a week after surgery **(Ca)** and again at 18 days after surgery **(Cb)** (sham *n* = 10; model *n* = 10; model + EX-4 *n* = 10; model + EX-4 + EX9-39 *n* = 5). **(Ca)** Two-way ANOVA with repeated measures (trials 1, 2, and 3) revealed that the pre-motor group differed in the progressive decline of the exploration time (s) for paprika [*F*_paprika(6,46)_ = 3.723, *P* < 0.05] 7 days after surgery. The pre-motor model did not display the classical decline in exploratory time following the three presentations of the social odors with an increase in the exploratory time at trial 3 and no response following the presentation of the Social 1 odor and no exploration of Social 2 odor. ^∗^*P* < 0.05, ^∗∗^*P* < 0.01, ^∗∗∗^*P* < 0.001, ^∗∗∗∗^*P* < 0.0001 for pre-motor baseline compared with pre-motor at 7 days after surgery. **(Cb)** At 18 days after surgery, the pre-motor model did not explore the vanilla odor during the trials 2 and 3 or the two social odors presented. EX-4 prevented the non-response following the first presentation of the social 1 and 2 odors. This effect was inhibited by the addition of EX9-39 (two-way ANOVA with repeated measures – ^∗∗∗^*P* < 0.001, ^∗∗∗∗^*P* < 0.0001 for pre-motor model at baseline compared with pre-motor at 18 days after surgery; & for pre-motor at 18 days after surgery compared with pre-motor + EX-4; $ for pre-motor + EX-4 compared with pre-motor + EX-4 + EX9-39; £ *P* < 0.05 for pre-motor model at baseline compared with pre-motor + EX-4).

General olfactory function was investigated using the hidden food test (Figure 4A). The pre-motor model displayed an increased latency to finding the treat compared with the sham animals at 18 days after surgery (one-way ANOVA with Gabriel’s method *post hoc* analysis, *P* < 0.05). Treatment with EX-4 prevented this increase (*P*_modelvs._ _model__+__EX-4_ <0.05) by activation of GLP-1Rs, as the latency displayed by animals treated with the double toxins, EX-4 and pre-treatment with EX9-39, was similar to that displayed by the pre-motor model. Open food tests with the treat in view were conducted to rule out a lack of motivation to find the treat and latencies were similar in the three experimental groups (*t*-test, *P* > 0.05 Figure 4B).

The habituation/dishabituation test was used to assess general olfactory function (measure of the exploratory time for each odor during each trial), odor memory (habituation) and odor discrimination (dishabituation) at 7 days (Figure 4Ca) and 18 days after surgery (Figure 4Cb). General olfaction was affected at 7 days after surgery in the model (Figure 4Ca). Exploration times differed for the odors paprika, vanilla and social 2. Odor memory was also impaired in the models as they did not present a progressive decline in the exploration time over three consecutive presentations of the social odors 7 days after surgery. At 18 days after surgery, experimental groups differed in the progressive decline of the exploration times for social 1 and social 2 [*F*_social__1(18,32)_ = 3.115, *P* < 0.01; *F*_social__2(18,34)_ = 3.087, *P* < 0.01]. Treatment with EX-4 increased the exploration time on the first trial for social 1 (*P* < 0.05) and this effect was mediated by GLP-1 receptor activation (*P* > 0.05) (Figure 4Cb). The ability to discriminate between odors (dishabituation) was evaluated by the increase in exploration time when a new odor was presented. The pre-motor model displayed reduced exploration time for the novel presentation of paprika and vanilla compared with sham animals (*P*_paprika_ <0.05; *P*_vanilla_ <0.05).

### Neuroinflammation Is Present in the PC of the Pre-motor PD Model

**FIGURE 5:**
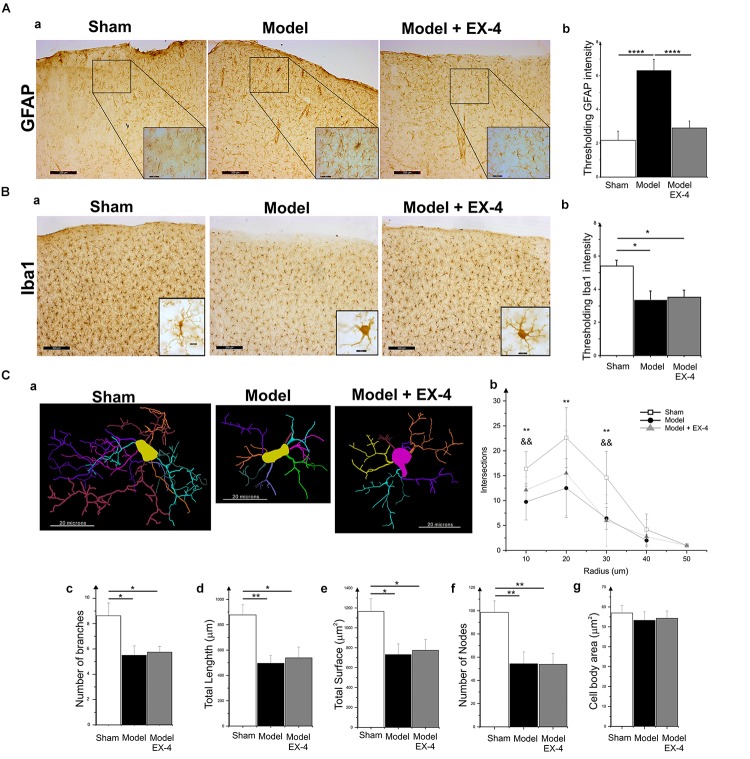
Neuroinflammation in the PC of the pre-motor PD model. **(A)** Astrocytic activation in the pre-motor PD model. **(Aa)** Representative immunohistochemical staining of GFAP in a sham animal, model and model treated with EX-4. Scale bars for overview and for inserts represent 200 and 50 μm, respectively. **(Ab)** Thresholding analysis of GFAP staining revealed an increase in the intensity in the pre-motor model compared with that in sham animals suggesting an activation of astrocytes. This increase was prevented by treatment with EX-4 (unpaired *t*-test, ^∗∗∗∗^*P* < 0.0001). Data are represented as mean ± SEM. **(B)** Microglia activation in the pre-motor PD model. **(Ba)** Representative immunohistochemical staining of Iba1 in a sham animal, model and model treated with EX-4. High magnification image of one Iba1-positive cell for each condition is represented in the inserts. Scale bars for overview and for inserts represent 200 and 10 μm, respectively. **(Bb)** Thresholding analysis of Iba1 staining revealed a decrease in the intensity in the pre-motor model compared with that in sham animals suggesting an activation of microglia. This decrease was not prevented by treatment with EX-4 (unpaired *t*-test, ^∗^*P*_shamvs._ _model__+__EX-4_ <0.05, *P*_modelvs.model__+__EX-4_ >0.05). Data are represented as mean ± SEM. **(C)** Morphological characteristics of Iba1-immunopositive cells. **(Ca)** Neurolucida reconstructions of Iba1-positive cells in the sham, model and model + EX-4. Each microglia branch is represented with a different color. Scale bars represent 20 μm. **(Cb)** Number of intersections between processes of Iba1-positive cells and concentric sphere at different radius from the soma in the sham, model and model treated with EX-4 (Sholl analysis). Data are represented as mean ± SD (*n* = 8 per experimental group – unpaired *t*-test – ^∗∗^*P* < 0.01 for pre-motor model compared with sham; && *P* < 0.01 for sham compared with pre-motor model treated with EX-4). **(Cc–g)** The number of branches **(Cc)**, total length **(Cd)**, total surface **(Ce)**, and the number of nodes **(Cf)** of Iba1-positive cells were decreased in the pre-motor PD model compared with those in the sham animals. Treatment with EX-4 did not prevent this decrease. The size of cell somata was similar in the sham, model and model + EX-4 **(Cg)**. Data are represented as mean ± SD (*n* = 8 per experimental group – unpaired *t*-test ^∗^*P* < 0.05, ^∗∗^*P* < 0.01).

Specific markers for astrocytic response, GFAP, and microglial activation, Iba1, were used to assess the presence of neuroinflammation in the PC of the pre-motor PD model (Figure 5). At 18 days after surgery, a stronger GFAP staining was observed in the PC of the model compared with that in the sham animals (Figures 5Aa,b; *P* < 0.0001). GFAP-positive astrocytes in all layers of the PC of the model displayed enlarged somata, extensive branching processes and overlapping domains (insert in Figure 5Aa). In contrast, a decrease in overall Iba1 staining intensity was observed in the PC of the pre-motor model compared with that in the sham animals at 18 days after surgery (Figures 5Ba,b; *P* < 0.05), reflecting a change in cell morphology (Figure 5C). Iba1-positive microglia displayed a decreased number of processes and less complex and shorter branches in the PC of the pre-motor model than in the sham animals, indicative of microglial activation (Figures 5Ca–f). The size of the somata in the model was, however, similar to that in the sham animals (*P* > 0.05; Figure 5Cg). Treatment with EX-4 reduced the astrocytic activation in the PC of the model (Figures 5Aa,b). However, EX-4 did not prevent microglial activation in the model (Figures 5Ba,b).

Interestingly, neuroinflammation in the PC of the model was already present a week after the stereotaxic injection and it followed a rostro-caudal gradient at both 7 and 18 days after surgery with a stronger staining observed in the anterior PC than in the medial PC and lighter staining in the posterior PC (Supplementary Figure S3). A stronger staining was observed at 18 days after surgery (Supplementary Figure S3).

### Down Regulation of Interneuronal Calcium Binding Proteins and Vasoactive Intestinal Polypeptide (VIP) Is Observed in the PC of the Pre-motor PD Model

**FIGURE 6:**
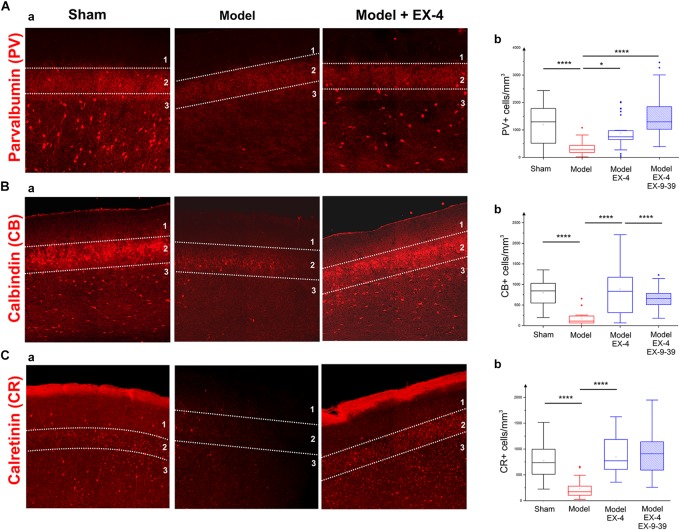
Distribution of calcium-binding proteins (CBPs) in the model of pre-motor PD. **(A)** Neuroprotective effect of EX-4 on parvalbumin (PV)-immunopositive interneurons in the PC of the pre-motor model. **(Aa)** Example of PV-immunostaining in the anterior PC of sham animals, model and model + EX-4 treatment at 19 days after surgery. **(Ab)** Cell densities in the PC of all experimental groups at 19 days after surgery expressed as the number of PV-positive neurones per mm^3^. The number of PV-positive interneurons was significantly decreased in the pre-motor model compared with that in the sham animals. Treatment with EX-4 prevented this decrease. The distribution of PV-positive neurones following treatment with EX-4 and EX9-39 was similar to that in sham animals. **(B)** Neuroprotective effect of EX-4 on calbindin (CB)-immunopositive interneurons in the PC of the pre-motor model. **(Ba)** Example of CB-immunostaining in the anterior PC of sham animals and model with and without EX-4 treatment at 19 days after surgery. **(Bb)** Cell densities in the PC of all experimental groups at 19 days after surgery expressed as the number of CB-positive neurones per mm^3^. A large decrease in the number of CB-positive interneurons was observed in the pre-motor model compared with the sham animals. Treatment with EX-4 prevented this decrease following activation of GLP-1Rs as the distribution of CB-positive neurones was similar to that in the pre-motor model. **(C)** Distribution of calretinin (CR)-immunopositive interneurons in the PC of the pre-motor model. **(Ca)** Example of CR-immunostaining in the anterior PC of sham animals and model with and without EX-4 treatment at 19 days after surgery. **(Cb)** Cell densities in the PC of all experimental groups at 19 days after surgery expressed as the number of CR-positive neurones per mm^3^. A decrease in the number of CR-positive interneurons was observed in pre-motor model compared with the sham animals. Treatment with EX-4 prevented this decrease. The distribution of CR-positive neurones following treatment with EX-4 and EX9-39 was similar to that in the sham animals and pre-motor model treated with EX-4 only (Kruskal–Wallis test ^∗^*P* < 0.05, ^∗∗^*P* < 0.01, ^∗∗∗^*P* < 0.001, ^∗∗∗∗^*P* < 0.0001).

To study the cellular structure of the PC in the pre-motor PD model, the distributions of different classes of interneurons were studied and compared with those in sham animals. Interneurons were classified according to their calcium-binding protein [parvalbumin (PV), calbindin (CB), calretinin (CR)] or peptide content [cholecystokinin (CCK), somatostatin (SOM), neuropeptide Y (NPY) and VIP]. Cell densities in the PC of all experimental groups at 19 days after surgery are presented in Table 2 and examples of the distributions of PV-, CB- and CR-immunopositive interneurons of the sham animals, pre-motor model and of the model treated with EX-4 at 19 days after surgery are displayed in Figures 6A–C. The number of PV, CB and CR- immunopositive interneurons in the PC of the pre-motor model was significantly reduced (Table 2 and Figures 6A–C Kruskal–Wallis test *P* < 0.05); VIP-immunopositive interneurons were also significantly reduced in number compared with the sham animals (Kruskal–Wallis *P* < 0.05) (Table 2). In contrast, the number of CCK-, SOM-, and NPY-immunopositive interneurons in the PC of all experimental groups was similar to that in the sham animals (Kruskal–Wallis test *P* > 0.05) (Table 2).

**Table 2:** Cellular densities of diverse interneuronal populations in the PC of the sham animals, sham animals treated with EX-4, pre-motor PD model and model treated with EX-4.

	SHAM + saline	SHAM + EX-4	6-OHDA + DSP-4 + saline	6-OHDA + DSP-4 + EX-4
GAD-67	2587.8 (2247.6–2927.9)	2808.9 (2067.5–3550.3)	2610.7 (1908.9–3312.5)	2178.0 (1742.1–2613.9)
PV	1294.6 (576.5–2012.7)	950.23 (514.2–1386.2)	278.7 ^∗∗∗∗^ (136.6–420.8)	749.1 #### (581.7–916.6)
CB	843.7 (591.3–1096.1)	1048.2 (668.2–1428.2)	110.6 ^∗∗∗∗^ (26.1–195.1)	834.2 #### (403.6–1264.8)
CR	736.6 (487.1–986.0)	727.3 (309.0–1145.6)	171.8 ^∗∗∗∗^ (80.2–263.3)	772.2 #### (473.8–1070.6)
CCK	172.2 (113.8–230.7)	195.7 (150.2–241.1)	139.7 (92.8–186.5)	164.8 (116.4–213.3)
SOM	619.7 (549.5–689.9)	414.4 (328.3–500.5)	439.8 (347.6–531.9)	436.0 (361.2–510.8)
NPY	173.7 (135.9–211.4)	202.2 (162.6.94–241.9)	167.6 (120.1–215.1)	145.8 (104.0–187.6)
VIP	472.8 (329.9–615.7)	424.9 (348.9–500.8)	224.5 ^∗∗∗^ (94.3–354.6)	482.6 ## (295.5–669.6)


*Data are represented as the median and interquartile range of the number cells per mm^3^ that are immunopositive for the indicated marker (*n* = 4–5 animals/per experimental group). GAD-67, glutamic acid decarboxylase-67; PV, parvalbumin; CB, calbindin; CR, calretinin; CCK, cholecystokinin; SOM, somatostatin; NPY, neuropeptide Y; VIP, vasoactive intestinal peptide (Kruskal–Wallis test was used, *post hoc* analysis for comparisons with Sham animals ^∗^*P* < 0.05; ^∗∗^*P* < 0.01; ^∗∗∗^*P* < 0.001; ^∗∗∗∗^*P* < 0.0001; *post hoc* analysis model compared with model treated with EX-4 ^#^*P* < 0.05; ^##^*P* < 0.01; ^###^*P* < 0.001; ^####^*P* < 0.0001).*

The observed loss of the PV, CB, CR, and VIP-immunopositive cells was prevented by treatment with EX-4 (Kruskal–Wallis *P* < 0.05), but surprisingly, pre-treatment with the GLP-1R antagonist EX9-39 only prevented the effect of EX-4 on the CB staining. Although the addition of EX9-39 did not completely prevent the protective effect of EX-4 (*P* > 0.05), the intensity of the PV- and CR-staining was decreased (Supplementary Figure S4). The study of the distributions of the PV, CB, and CR interneurons in the toxin models prior to EX-4 treatment (at 8 days after surgery) revealed no significant changes in neurone numbers relative to sham controls (Kruskal–Wallis test *P* > 0.05; Supplementary Figure S5).

**FIGURE 7:**
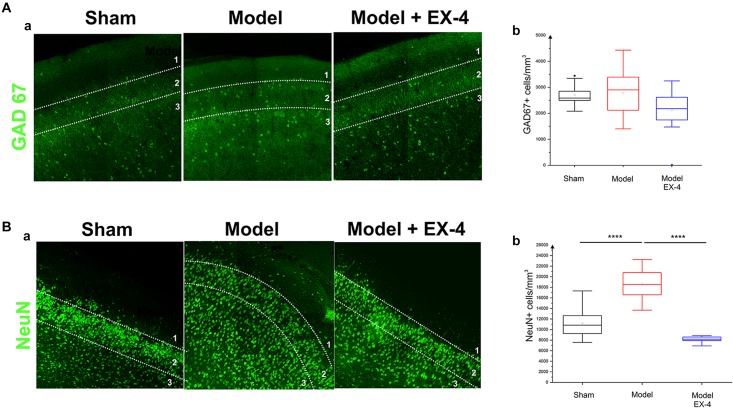
Distribution of NeuN- and GAD-immunopositive neurones in the model of pre-motor PD. **(A)** Distribution of GAD-67-immunopositive interneurons in the PC of the pre-motor model. **(Aa)** Example of GAD-67-immunostaining in the anterior PC of sham animals, model and model + EX-4 treatment at 19 days after surgery. **(Ab)** Cell densities in the PC at 19 days after surgery expressed as the number of GAD-67-positive neurones per mm^3^. The distributions were similar in all experimental groups. **(B)** Distribution of NeuN-immunopositive interneurons in the PC of the pre-motor model. **(Ba)** Example of NeuN-immunostaining in the anterior PC of sham animals and models with and without EX-4 treatment at 19 days after surgery. **(Bb)** Cell densities in the PC at 19 days after surgery are expressed as the number of NeuN-positive neurones per mm^3^ ± SEM. The density of NeuN-positive neurones in the pre-motor model increased compared with that in the sham animals. This increase was prevented by treatment with EX-4. ^∗∗∗∗^*P* < 0.0001.

The number of GAD-67-immunopositive neurones was also studied in the PC of all experimental groups to determine whether the striking changes observed in the PC of the model was due to a down-regulation of the calcium-binding proteins and/or VIP or to loss of neurones. No significant difference was found between the densities or distributions of GAD-67-positive cells in the sham animals, in the pre-motor models and in the models treated with EX-4, suggesting a down regulation of the calcium-binding proteins PV, CB, CR, and peptide VIP in the model rather than loss of cells (Table 2 and Figures 7Aa,b).

### Increased Number of NeuN-Immunopositive Cells in the PC of the Pre-motor PD Model

To further study the cellular structure of the PC in the pre-motor PD model, the distribution of NeuN-immunopositive neurones was observed in the PC of the sham animals and pre-motor model at 18 days after surgery, revealing an increase in the number of neurones in the model (Figures 7Ba,b – *P*_shamvs._ _model_ <0.0001) that resulted mainly from an increase in the number of neurones in Layer 3 of the PC (data not shown). This increase was not apparent at 8 days after surgery (Supplementary Figure S5) and after treatment with EX-4 (Figures 7Ba,b – *P*_modelvs._ _model__+__EX-4_ <0.0001).

### Degradation of the Perineuronal Nets (PNNs) in the PC of the Pre-motor PD Model

**FIGURE 8:**
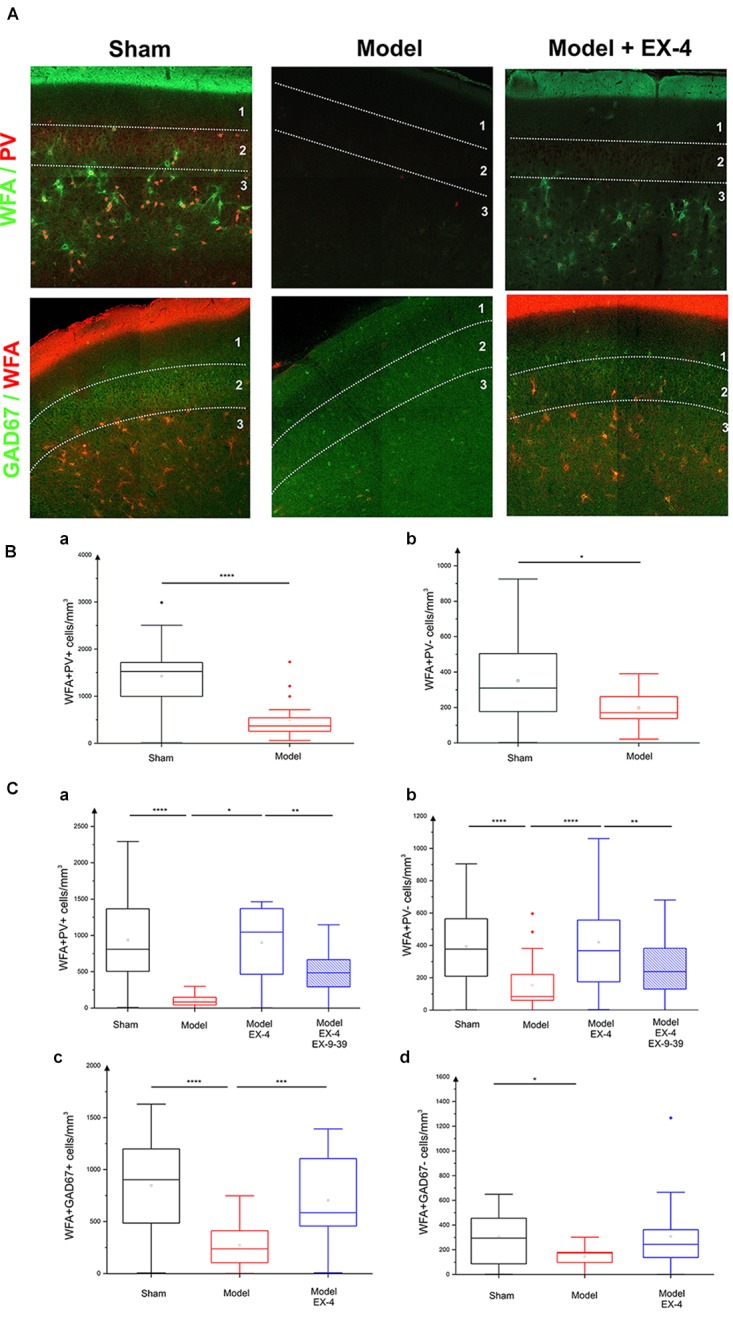
Effect of EX-4 on the distribution of perineuronal nets (PNNs) in the PC of the pre-motor model. **(A)** Slices of the anterior PC of sham animals, model treated either with saline or EX-4 were double stained for PNNs (WFA in green) and PV (in red) (top row) and for PNNs (WFA in red) and GAD-67 (in green) (bottom row) at 19 days after surgery. **(B)** Cell densities in the PC of the sham animals and toxin-treated animals at 8 days after surgery expressed as the number of WFA-positive and PV-positive neurones per mm^3^ **(Ba)** and as the number of WFA-positive and PV-negative neurones per mm^3^ **(Bb)**. A decrease in the number of both WFA+/PV+ and WFA+/PV– cells was observed compared with that in the sham animals and models. **(Ca)** Densities of WFA+/PV+ neurones in the PC of the pre-motor model were decreased compared with that in sham animals at 19 days after surgery. The decrease was prevented by treatment with EX-4 and the addition of EX9-39 inhibited the effect of EX-4 in the model. **(Cb)** Densities of WFA+/PV– neurones in the PC of the model were also decreased compared with that in sham animals at 19 days after surgery. EX-4 prevented the decrease in the number of WFA+/PV– cells. Densities of WFA+/GAD+ neurones **(Cc)** and WFA+/GAD– neurones **(Cd)** in the PC at 19 days after surgery were decreased in the model compared with that in the sham animals. Treatment with EX-4 prevented the decrease of WFA+/GAD+ neurones only (Kruskal–Wallis test ^∗^*P* < 0.05, ^∗∗^*P* < 0.01, ^∗∗∗^*P* < 0.001, ^∗∗∗∗^*P* < 0.0001).

In view of the dramatic effects on PV interneurone staining seen in the pre-motor model described above and the previously described link between PNNs and PV neurones (Berretta et al., 2015), the effects of the toxin injections on the integrity of the PNNs in the PC in the pre-motor model were studied. Sections were double labeled with the lectin Wisteria Floribunda Agglutinin (WFA) and PV to determine whether the loss of calcium-binding proteins was associated with a loss of PNNs. Examples of the WFA+/PV+ staining of neurones in the anterior PC of the sham animals, pre-motor PD model and model treated with EX-4 are displayed in the top panels in Figure 8A. The numbers of PV+/WFA+ neurones at day 8 and 19 after surgery are presented in Figures 8Ba,Ca. A significant decrease in the number of PV+/WFA+ neurones was observed in the PC of pre-motor model at 8 days after surgery when compared with sham animals (*P* < 0.0001) (Figure 8Ba). A further decrease was observed at 19 days after surgery (*P* < 0.0001) (Figure 8Ca). Treatment with EX-4 prevented the loss of both PV+ and WFA+ neurones in the pre-motor model (*P* < 0.05) (Figure 8Ca). Pre-treatment with the GLP-1R antagonist EX9-39 partially blocked the protective effects of EX-4 treatment as assessed by PV+/WFA+ staining in the pre-motor PD model (*P* < 0.01) (Figure 8Ca and Supplementary Figure S4).

The PC also contained a small population of PV-immunonegative (PV-) cells that were also surrounded by PNNs. A decrease in PV-/WFA+ cells in the PC of the pre-motor model was observed at 8 days (Figure 8Bb) and at 19 days after surgery (Figure 8Cb). Treatment with EX-4 prevented the loss of these PV-/WFA+ cells (*P* < 0.0001). Finally, pre-treatment with EX9-39 partially blocked the effects of EX-4 treatment in the pre-motor PD model (*P* < 0.01) (Figure 8Cb and Supplementary Figure S4). The WFA-immunopositive neurones were shown to be either GABAergic (see GAD-67+/WFA+ staining in bottom panels in Figures 8A,Cc) or GAD-67-immunonegative (Figure 8Cd). Treatment with EX-4 prevented the loss of GAD-67+/WFA+ cells only (*P* < 0.001) (Figure 8Cc).

## Discussion

Detection of the loss of smell (hyposmia) in early-stage PD patients (potentially as early as 10 years before diagnosis) may greatly facilitate a clinical diagnosis and allow neuroprotective-based treatments to commence that could slow the progression of the disease and delay the onset of more debilitating PD symptoms. However, the pathophysiological changes behind hyposmia remain to be further characterized. Despite a large number of studies on olfactory dysfunction in late stage PD, none have specifically examined any possible structural changes in the piriform cortex region. To address this, we used a dual neurotoxin-based model of pre-motor PD that displays non-motor symptoms in the absence of motor symptoms, to understand the etiology of hyposmia and specifically to detect any changes in this region that may underlie this early symptom. Neuroinflammation and PNN disruption were present in the PC of the pre-motor PD model a week after toxin induction and these changes were followed by a marked decrease of CBPs and VIP in PC interneurons. Importantly, most of the observed structural changes were prevented by EX-4 treatment, through GLP-1R activation.

### The Model of Pre-motor PD Displayed Olfactory and Cognitive Deficits in the Absence of Motor Dysfunction

Parkinson’s disease has been described as a multisystem disorder that affects several body systems and neurotransmitters (Beach et al., 2010; Klingelhoefer and Reichmann, 2017). The “dual-hit” hypothesis described by Braak and colleagues (Braak et al., 2003, 2004; Hawkes et al., 2007; Braak and Del Tredici, 2017) proposed that a pathogen or virus is able to enter the brain through two routes, the nose and the gut, leading to LP. The pathology is then retrogradely spread through the brain via synaptic connections reaching first the LC (stage 2), then the SNc where it targets dopaminergic neurons resulting in the hallmark motor symptoms of the pathology (stage 3), and later the neocortex (stage 6). However, while the Braak staging theory is accepted by most, the LP distribution in a large number of patients, does not follow this ascending progression (Kalaitzakis et al., 2008; Halliday et al., 2012) suggesting a selective neuronal vulnerability based on their long-range connectivity, distinct physiological properties and/or functional threshold (“threshold” theory: Surmeier et al., 2017; Engelender and Isacson, 2017). Accordingly, the early appearance of NMS in the prodromal phase of the disease may be explained by a low “threshold” of the peripheral, autonomic and enteric systems leading to a lack of compensatory mechanisms to maintain their functions and therefore an increased vulnerability of these regions (Engelender and Isacson, 2017). The pathophysiological changes behind the emergence of these NMS is, however, poorly understood and it is still a matter of debate whether the depletion of neurotransmitters rather than the early accumulation of α-synuclein in the olfactory system is responsible for the olfactory dysfunction in PD patients (Doty, 2017). While the induction of the pre-motor model used in this study does not initiate the accumulation of α-synuclein in brain regions affected in early stage PD, this model was specifically used to study the effect of the *partial* loss of NAergic and DAergic cells in the LC and SNc and consequently of the reduction of the neurotransmitter levels in these and connected regions. Importantly, the dual toxin-based model of pre-motor PD enabled the investigation of the early onset NMS in the absence of motor signs. Injections of both DSP-4 and 6-OHDA resulted in a clear decrease in the number of TH-immunopositive cells in the LC and SNc, respectively. Dopaminergic and noradrenergic lesions resulted in a decrease of ∼50% of DA and NA levels in the SNc of the pre-motor model and behavioral assessments revealed that significant olfactory and cognitive deficits were displayed by the model without anhedonia or motor dysfunction. This suggests that this model may represent an earlier stage of the disease than in previously described animal models in which anxiety and depression-like symptoms, cognitive deficits or olfactory dysfunctions were reported in the presence (albeit limited) of motor dysfunctions (Tadaiesky et al., 2008; Santiago et al., 2010; Carvalho et al., 2013; Costa et al., 2014; Faggiani et al., 2015; Ledreux et al., 2016; Nezhadi et al., 2016). The development of animal PD models focusing mainly on the nigrostriatal DAergic system has provided valuable insight into the etiology of motor symptoms of PD for many decades; however, each model has its own strengths and limitations and none of them recapitulates all the symptoms of the disease exactly (Grandi et al., 2018; Vingill et al., 2018). While a loss of DA results in a loss of motor function in these models, non-motor symptoms of PD cannot be accounted for by the DA depletion alone, suggesting an implication of other neurotransmitters, including NA, in the etiology of these deficits (Delaville et al., 2011). Although impairments of both DAergic and NAergic systems have been shown in the human disease (Jenner et al., 1983; Gaspar et al., 1991; Buddhala et al., 2015; Nahimi et al., 2018; Peterson and Li, 2018), the implication of NA in the disease progression has often been ignored, hence the focus on DA depletion alone in animal models (with addition of uptake inhibitors to prevent the effect of 6-OHDA on NAergic cells in most studies). The pre-motor model used in this study displayed a moderate NA depletion combined with a moderate DA loss that resulted in non-motor symptoms (hyposmia and cognitive deficits) in the absence of motor deficit 24/25 days after the injection of DSP-4. The combination of the depletions was essential for the appearance of olfactory deficits in the pre-motor PD model, as depletion of either DA alone or NA alone (using the same low toxin concentrations) was not sufficient to elicit hyposmia in both hidden and habituation/dishabituation tests in the treated animals (data not shown). This is in agreement with previous studies that show PD patients with dopamine beta-hydroxylase (DBH) deficiency displaying a normal olfactory function (Garland et al., 2011) and an intact odor detection performance following NA depletion in the OB (Doty et al., 1988).

Hyposmia, characterized by the inability to perceive, recognize, and discriminate or memorize odors, occurs in ∼90% of PD patients (Doty et al., 1988; Mesholam et al., 1998; Berendse et al., 2011; Doty, 2012). It is thought to be an early event occurring several years prior to clinical diagnosis and to be a good predictor of cognitive decline in neurodegenerative diseases and therefore a reliable aid to early PD diagnosis (Devanand et al., 2010; Berendse et al., 2011; Doty, 2012, 2017). Impaired olfactory function and a reduced ability to learn odors (shown by impaired habituation), indicative of cognitive deficits, were observed in our model as early as 7 days after toxin-injection. These olfactory deficits were unlikely to have resulted from reduced motivation, since the number of TH-immuno-positive cells in the VTA (a dopaminergic brain area known to be involved in cognition, motivation and emotion) was not affected by the 6-OHDA toxin injection and the animals were able to locate visible treats during an open food test. Like PD patients (Doty, 2012), the rats were hyposmic, but not anosmic, as animals found the treat in the hidden food test and displayed exploratory activity in the habituation/dishabituation test. We cannot preclude a role of the cognitive deficit on olfaction as odor detection, identification, discrimination or memory rely upon each other for a normal olfactory function. However, the striking PC structural changes, observed in this study, may also be responsible for the compromised olfactory function observed in the pre-motor model.

### Role of Noradrenergic and Dopaminergic Inputs Into the Piriform Cortex in the Early Stage of PD

Poor olfactory performance in PD patients has been associated with a volume reduction in the olfactory bulbs (Brodoehl et al., 2012; Altinayar et al., 2014; Li et al., 2016) and PC (Wattendorf et al., 2009; Chen et al., 2014; Lee et al., 2014) and the severity of the disease correlates directly with the severity of the deficit in the PC (Wattendorf et al., 2009). A profound hyperactivation in the PC of hyposmic PD patients, subjected to an odor detection task and the consequent network dysfunction (Moessnang et al., 2010) may be attributed to the loss of dopaminergic and noradrenergic inputs to the PC, as shown in this model (with a 40% loss of both neurotransmitters) and in a previous study (Becker et al., 2018), that would normally have local “dampening” effects by increasing interneuronal spontaneous activity (Gellman and Aghajanian, 1993; Hasselmo et al., 1997; Giorgi et al., 2003, 2006).

The existence of long-range noradrenergic innervation in the piriform cortex and its influence on synaptic plasticity/neuronal function is indeed now well established (Fallon and Moore, 1978; Constanti and Sim, 1987; Bouret and Sara, 2002; Ghosh et al., 2015; Vadodaria et al., 2017). Likewise, dopaminergic projections into the PC may be important in olfactory-guided learning (Garske et al., 2013) as well as the maintenance of PV-expressing interneurons and pyramidal neurone morphology (Stanwood et al., 2001). In addition to these important synaptic functions, there is now considerable evidence favoring a general intrinsic neuroprotective role for these neurotransmitters in the brain (noradrenaline: O’Neill and Harkin, 2018; dopamine and dopamine receptor agonists: Schapira, 2002; Olguín et al., 2018; Tozzi et al., 2018). Dopamine agonists also possess anti-α-synuclein protein aggregation properties (Luo et al., 2016; Yedlapudi et al., 2016). The neuroprotective effects of dopamine agonists accord well with their now well-established use in the treatment of early Parkinson’s disease (Marsili et al., 2017) and could involve a variety of underlying mechanisms including antioxidation, ROS scavenging and inhibition of apoptosis (Bonuccelli and Pavese, 2007). Thus, it is reasonable to propose that the neuroinflammation, structural and interneuronal calcium binding protein changes we observed in the PC following dual toxin treatment, were the direct result of a reduction in background neuroprotection normally offered by noradrenergic and dopaminergic inputs into this area. The consequent interference with local circuit function could then be responsible for the behavioral changes in olfaction that were characteristic of our model, which were effectively prevented by EX-4 treatment.

### Neuroinflammation Is Present in the PC of the Pre-motor PD Model

Hyposmia observed in the model of pre-motor PD was associated with striking changes in the PC, including neuroinflammation and cellular changes. Activated astrocytes were observed in the PC of the model of pre-motor PD at 19 days after surgery. Observation of the GFAP staining also revealed that an increased level of neuroinflammation in the PC of the pre-motor model compared with that in sham animals was already present at 8 days after surgery with a stronger staining in the anterior PC. Activation of astrocytes was progressive in nature and followed a rostro-caudal gradient suggesting that glial cells in the anterior PC may therefore activate neighboring cells via gap junctions, with the release of toxic pro-inflammatory factors as previously suggested (Lucin and Wyss-Coray, 2009) leading to the progression of neuroinflammation across the PC region. An apparent decline in Iba1 overall intensity in the PC of the pre-motor model was also observed. This decline was due to changes in cell morphology, including a decreased number of protruding branches and shorter processes, typical of microglial activation (Block and Hong, 2005; Wang et al., 2015).

Neuroinflammation has been shown to be involved in PD pathogenesis with the synergic activation of both astrocytes and microglia contributing to an enhanced death of DAergic neurones in the SNc (Glass et al., 2010; Iannaccone et al., 2013; Wang et al., 2015). The cause of neuroinflammation and the specific roles of astrocytes and microglia in the development of the disease remain poorly understood. However, both DA-mediated toxicity and the release of pro-inflammatory cytokines released by activated microglia and astrocytes are thought to play a vital role in the degeneration of DA neurones (Rabinovic et al., 2000; Ferrari et al., 2006; Caudle et al., 2007; Lecours et al., 2018). As the disease progresses, these degenerative neurones then amplify the inflammatory responses and the process of neurodegeneration by releasing molecules such as α-synuclein and adenosine triphosphate (ATP) that further enhance microglia activation (Davalos et al., 2005). The presence of neuroinflammation in the PC of the pre-motor model may also potentially be attributed to the decrease in NAergic inputs to this region as NA has the ability to suppress microglial pro-inflammatory activity through activation of microglial adrenergic receptors; furthermore, reduced levels of NA have been associated with progressive neuronal degeneration in both AD and PD patients (Heneka et al., 2010; Kong et al., 2010; Vermeiren and De Deyn, 2017; O’Neill and Harkin, 2018).

### Are Cellular Changes Observed in the PC, Potential Landmarks for Pre-motor PD?

This study examined the pathophysiological changes occurring in the olfactory cortex of a PD model for the first time. Striking cellular changes were observed following both noradrenergic and dopaminergic lesions. Interestingly, the number of NeuN-immunopositive neurones *increased* in the PC of the pre-motor PD model compared with that in the sham animals. Although the cause of this increase remains unknown, it is possible that structural changes observed in the PC of the model caused by degradation of the PNNs resulted in a reorganization of neurones in this brain area. PNNs are highly condensed ECM aggregates that form honeycomb structures around PV-positive interneurons in several brain regions including the PC (Alpár et al., 2006; Ajmo et al., 2008) and around glutamatergic neurones in the parietal cortex, the hippocampal CA2 region, the amygdala and PC (Härtig et al., 1999; Brückner et al., 2004; Carstens et al., 2016; Morikawa et al., 2017). We can hypothesize that the disruption of the PNN and therefore the extracellular matrix may result in the migration of the cells away from their original laminar location. Although a disruption of lamination has yet to be described in PD patients to date, such a disruption has been associated with epilepsy (Chae et al., 1997). Reeler is a transgenic mouse model caused by the autosomal recessive genetic mutation in reelin – a secretory ECM protein (D’Arcangelo et al., 1997). The disruptive lamination in this mouse model is the result of defective formation of the CNS, whilst the CNS in the pre-motor PD model was intact prior to toxin administration. However, the possible link between a disrupted lamination, deficits in the ECM and occurrence of epilepsy found in previous studies (Saghatelyan et al., 2001; Dityatev et al., 2007, 2010) may suggest that the loss of distinct PC layers in the pre-motor PD model is the result of an ECM disruption.

This PNN degradation was observed at 8 days after surgery and coincided with the presence of activated astrocytes in this region. Although the cause of the PNN degradation is unknown, one could speculate a role of inflammation in this process (Baig et al., 2005; Hobohm et al., 2005). The loss of PNNs may reflect a defect in the ability of glial cells to secrete vital PNN components, e.g., chondroitin sulfate proteoglycans (CSPGs) (Faissner et al., 2010; Klausmeyer et al., 2011; Wiese et al., 2012). However, whether the secretion and production of extracellular matrix proteins by activated glia is altered under chronic inflammatory conditions in the pre-motor model has yet to be determined. Alternatively, degradation of PNNs in the PC may result from the secretion of matrix metalloproteinase-9 (MMP-9) and a disintegrin-like and metalloproteinase with thrombospondin motifs (ADAMTS) from reactive astrocytes and neurones as shown in an epilepsy model (Pollock et al., 2014; Dzyubenko et al., 2016). Inflammatory microglial activation may also lead to degradation of the PNNs (Franklin et al., 2008).

Although the role of PNNs surrounding PC pyramidal cells is undetermined, PNNs have been shown to restrict synaptic plasticity in the hippocampal CA2 pyramidal cells (Carstens et al., 2016). Loss of PNNs may also render interneurons more susceptible to oxidative stress due to astrocytic activation as early as 8 days after surgery, potentially leading to a reduction of PV mRNA expression and a potential disruption of oscillatory activity as previously shown in the hippocampus (Yamada et al., 2015; Sun et al., 2018). The number of GAD-immunopositive neurones in the PC of the pre-motor model was similar to that in the sham animals, suggesting a downregulation of CBPs rather than neuronal death. The dramatic decrease in the CBP expression in the PC (mimicking the vulnerability of these cells in olfactory regions of PD patients; Ubeda-Banon et al., 2010; Doty, 2012), will eventually lead to a disruption of calcium buffering and cell death due to calcium toxicity. The downregulation of CBPs is likely to modulate the firing of interneurons in the PC of the pre-motor model and their targets (Caillard et al., 2000; Volman et al., 2011), potentially disturbing the excitation/inhibition balance in this region and ultimately altering behavioral performance on smell identification/discrimination as previously shown in Alzheimer’s disease and PD patients (Li et al., 2010; Moessnang et al., 2010). The characterization of potential compensatory mechanisms in other brain regions as a consequence of these changes, was beyond the scope of this study and remains to be explored.

Further studies are also needed to determine the timing of the observed changes. Although the number of PV- and CB-immunopositive cells and VIP-positive cells was similar to that in the sham animals at 8 days after surgery, changes may have occurred earlier, but not have been visible until the system had failed to replace the existing CBPs. Similar considerations may explain the differential effect of the antagonist EX9-39 on the number of PV- and CR-positive neurones compared with that on CB-positive interneurons. Although the number of PV- and CR-neurones after treatment with EX9-39 was similar to that with EX-4, the intensity of the staining was lower, possibly suggesting a slower downregulation of these calcium binding proteins that did not result in a visible decrease in the number of neurones in the time course of the protocol used in this study.

### EX-4, a Promising New Treatment for Pre-motor PD?

The pre-motor model of PD provided further understanding of the early pathology of PD and a means to test a potential new therapy, EX-4. The presence of GLP-1 – producing cells in the OB and the known presence of EX-4 target, GLP-1Rs, in the PC (Cork et al., 2015; Thiebaud et al., 2016) led us to investigate the effect of EX-4 on the striking cellular changes observed in this region following dual neurotoxin treatment. Injections of EX-4 in sham animals had no effect on the animal behaviors (Supplementary Figure S6), level of inflammation or PC neuronal distributions (Table 2). However, EX-4 administration in the pre-motor model of PD prevented astrocytic activation, olfactory and cognitive impairments, the loss of PNNs and the reduction of some CBPs and VIP expression in the PC, through GLP-1R activation, since the effects were prevented by the competitive GLP-1R antagonist EX9-39.

EX-4 has been found to exert powerful neuroprotective properties in several other experimental neurodegenerative disease model systems including PD and Alzheimer’s disease (for review see Athauda and Foltynie, 2016a,b; Athauda and Foltynie, 2018), Huntington’s disease (Martin et al., 2009), amyotrophic lateral sclerosis and stroke (Hölscher, 2014) and other neuroinflammatory diseases (multiple sclerosis: Lee et al., 2018). However, the mechanism(s) by which EX-4 and other GLP1-R agonists exert these effects is still unclear. It has been suggested that such neuroprotective effects as well as general cell-protective effects on other cell systems (pancreatic β-cells: Li et al., 2013; adipocytes: Wang et al., 2017; Góralska et al., 2017; cardiomyocytes: Chang et al., 2018; hepatic cells) could be exerted via an improvement of mitochondrial function compromised by oxidative stress [reactive oxygen species (ROS) production]. The molecular defense mechanisms involved are likely to be varied and complex in different cells, however, sustaining mitochondrial membrane potential to prevent mitochondrial apoptosis and ultimate cell death could be a common factor governing effectiveness (Cunha et al., 2009). Of particular interest and relevance to the present study was a report by Song et al. (2018) showing that depletion of brain noradrenaline by DSP-4 treatment in mice, was linked to neuroinflammation, ROS production and subsequent neurodegeneration; moreover, it was proposed that nicotinamide adenine dinucleotide phosphate (NADPH) oxidase-2 (NOX2) was involved in the inflammation-mediated production of superoxide. The link between chronic neuroinflammation, microglial activation, oxidative stress (ROS production), mitochondrial dysfunction and PD pathophysiology was recently reviewed by Hassanzadeh and Rahimmi (2018), in which it is suggested as a general strategy, that alternative antioxidative and anti-inflammatory treatments for PD could replace or supplement existing therapies in the near future. We would advocate that use of EX-4 or other GLP-1R agonists could be part of this alternative treatment strategy, with the potential to improve the long-term prognosis for millions of PD patients world-wide.

To summarize, the model of pre-motor PD we have investigated, exhibited clear hyposmia, a recognized early characteristic of clinical PD. Olfactory deficits were associated with the presence of neuroinflammation in the PC, potentially leading to a disruption of PNNs and eventually a downregulation of CBPs and VIP from distinct local interneurons. The fact that these effects were prevented by EX-4 treatment indicates that this model may provide a means for research into early PD treatment. The future investigation of a GLP-1 therapeutic target system may lead to the possibility of earlier intervention for PD patients and a delay of disease progression.

## Ethics Statement

This study was carried out in accordance with the recommendations of UCL Bloomsbury AWERB. The protocol was approved by the Home Office United Kingdom.

## Author Contributions

MS and ES performed the experiments, and collected and analyzed the data. GE helped with the cell counting. MS and AM prepared the figures. AC and AM designed the study. AM wrote the manuscript draft. AC revised the manuscript.

## Conflict of Interest Statement

The authors declare that the research was conducted in the absence of any commercial or financial relationships that could be construed as a potential conflict of interest.

## Supplementary Material

Figure S1 (A) Representative images of TH-staining in the locus coeruleus (LC) in the sham-operated animals (left panel), pre-motor PD model (middle panel), and model treated with EX-4 (right panel). Scale bars represent 200 μm. **(B)** Number of TH-positive cells in the LC expressed as a percentage of sham (*n* = 4 animals per experimental group). The DPS-4 injections resulted in a decrease in the number of TH-positive cells in the LC of pre-motor model or model treated with EX-4 (unpaired *t*-test *P* < 0.05). This decrease was prevented by treatment with EX-4 (unpaired *t*-test *P* < 0.05). ^∗^*P* < 0.05; ^∗∗∗^*P* < 0.001.

Figure S2 (A) Representative images of TH-staining in the ventral tegmental area (VTA) in the sham-operated animals (left panel), pre-motor PD model (middle panel), and model treated with EX-4 (right panel). Scale bars represent 500 μm substantia nigra pars compacta (SNc). **(B)** Number of TH-positive cells in the VTA expressed as cell density per mm^3^ (*n* = 6 animals per experimental group). The double toxin injections had no effect on the number of TH-positive cells in the VTA of pre-motor model or model treated with EX-4 (unpaired *t*-test *P* > 0.05).

Figure S3 GFAP-positive activated astrocytes follow a rostro-caudal gradient in the piriform cortex (PC) of the pre-motor PD model at both 8 days **(A–C)** and 19 days **(D–F)** after surgery with a stronger staining in the anterior PC **(A,D)** than in the medial **(B,E)**, and posterior **(C,F)** regions. At 8 days after surgery, activated GFAP+ astrocytes, characterized by overlapping astrocytic domains (insert in **A**) were only found in the anterior PC **(A)** and the GFAP-immunopositive cortical surface was more intensely stained in the anterior PC than in the medial **(B)** and posterior PC **(C)**. GFAP staining at 19 days after surgery was stronger in all PC regions compared with that at 8 days after surgery and overlapping astrocytic domains were observed in the anterior (insert in **D**) and medial (insert in **E**) PC of the pre-motor model.

Figure S4 Example of PV-, CB-, CR-, and PNN+/PV+-immunostaining in the anterior PC of the model of pre-motor PD treated with EX-4 and GLP-1 antagonist EX9-39 at 19 days after surgery. Pre-treatment with the GLP-1R antagonist EX9-39 only prevented the effect of EX-4 on the CB staining. Although the addition of EX9-39 did not completely prevent the protective effect of EX-4 (*P* > 0.05), the intensity of the PV- and CR-staining was decreased. Pre-treatment with the GLP-1R antagonist EX9-39 partially blocked the protective effects of EX-4 treatment as assessed by PV+/WFA+ staining in the pre-motor PD model.

Figure S5 (A) Density of NeuN-positive neurones in the sham and pre-motor PD model at 8 days after surgery. **(B)** Density of CR-immunopositive neurones in the sham and toxin-treated animals at 8 days after surgery. **(C)** Density of PV-immunopositive neurones in the sham and toxin-treated animals at 8 days after surgery. **(D)** Density of CB-immunopositive neurones in the sham and toxin-treated animals at 8 days after surgery. The number of PV-positive neurones remained unchanged in all experimental groups. Cell densities are expressed as the number of immune-positive neurones per mm^3^ ± SEM (Kruskal–Wallis test was used the number of neurones remained unchanged between the two groups).

Figure S6 Effect of EX-4 injections on sham animals. **(A)** Cognitive function was tested in the sham animals treated with EX-4 only (*n* = 4). There was no change in the DR in these animals compared with sham animals (unpaired *t*-test *P* > 0.05). **(B)** Sham rats treated with EX-4 did not display anhedonia (*n* = 4 unpaired *t*-test *P* > 0.05). **(C,D)** Hyposmia in the sham animals treated with EX-4 was tested using the hidden food **(C)** and habituation and dishabituation **(D)** tests (*n* = 10). The latency time (s) taken by the sham animals treated with EX-4 to find the hidden treat was similar to that taken by the sham animals (unpaired *t*-test *P* > 0.05). Exploration times (s) following the three presentations of all odors were similar in the sham and sham treated with EX-4 (unpaired *t*-test *P* > 0.05). **(E)** Representative immunohistochemical staining of GFAP in a sham animal treated with EX-4. Inserts represent high magnification of one astrocyte. Scale bar for overview represents 200 μm.
