# Supplementary figures and images for: Structural Changes Observed in the Piriform Cortex in a Rat Model of Pre-motor Parkinson’s Disease
Source: Front Cell Neurosci. 2018 Dec 10;12:479. doi: 10.3389/fncel.2018.00479 (PMC6296349; doi:10.3389/fncel.2018.00479)

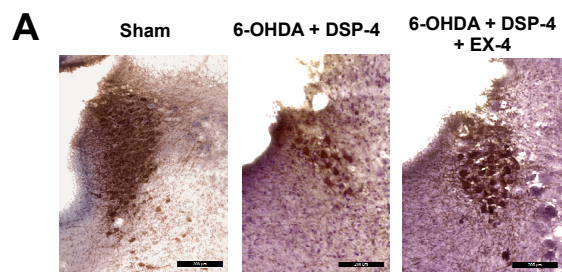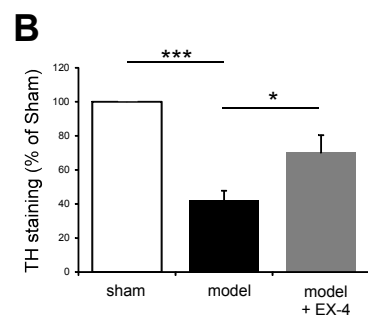

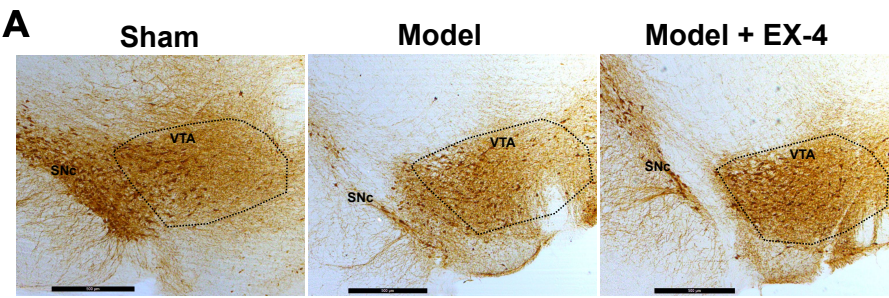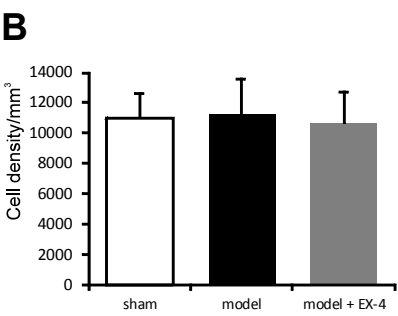

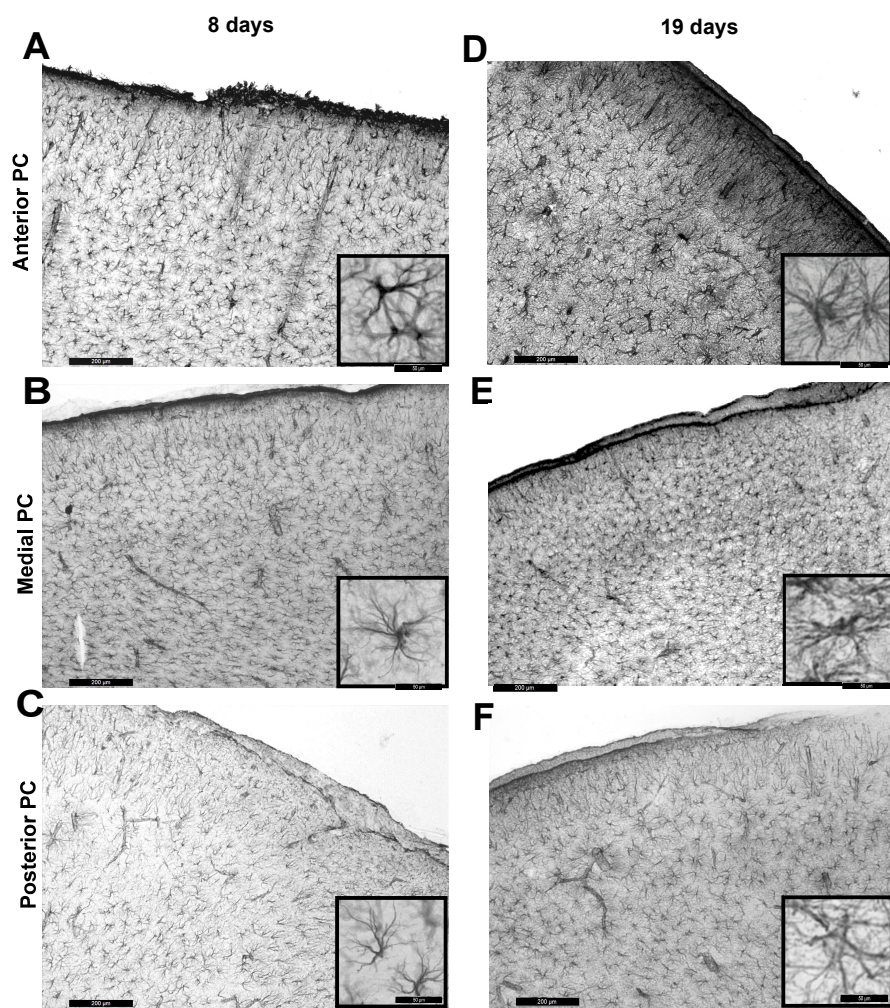

Model + EX-4 + EX9-39

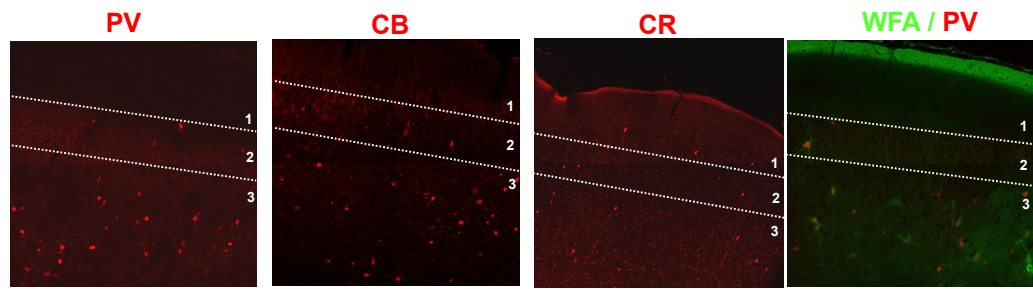

**A**

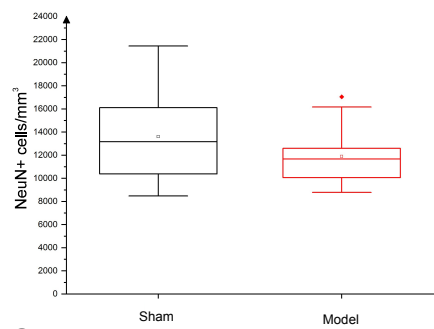

**B**

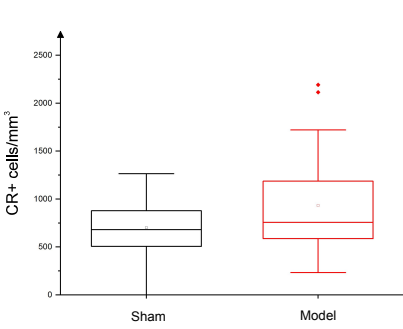

**C**

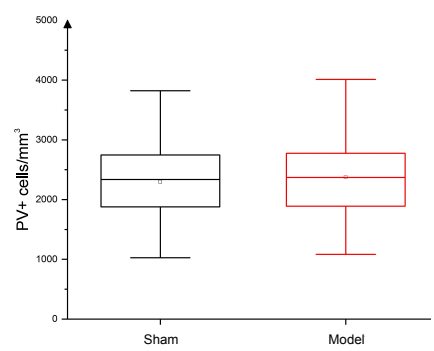

**D**

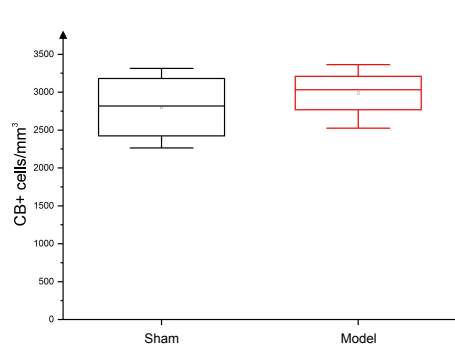

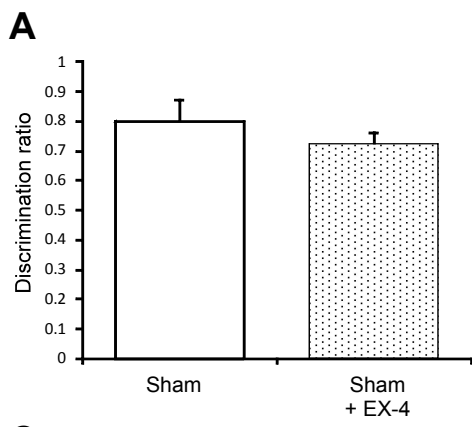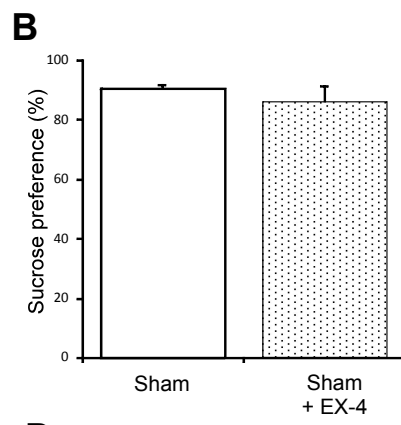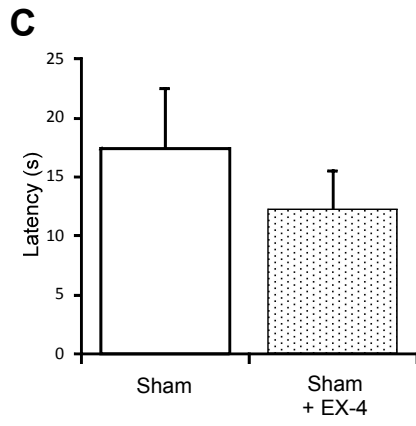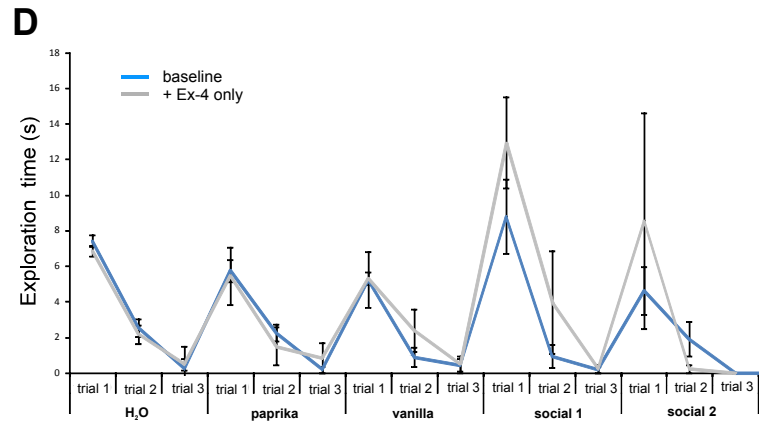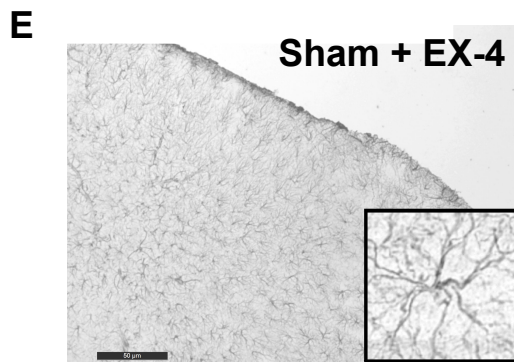

Supplement: Figure S1 — (A) Representative images of TH-staining in the locus coeruleus (LC) in the sham-operated animals (left panel), pre-motor PD model (middle panel), and model treated with EX-4 (right panel). Scale bars represent 200 μm. (B) Number of TH-positive cells in the LC expressed as a percentage of sham (n = 4 animals per experimental group). The DPS-4 injections resulted in a decrease in the number of TH-positive cells in the LC of pre-motor model or model treated with EX-4 (unpaired t-test P < 0.05). This decrease was prevented by treatment with EX-4 (unpaired t-test P < 0.05). ∗P < 0.05; ∗∗∗P < 0.001. [file Data_Sheet_1.pdf]
